# Effect of Parent-Mediated Home-Based Rehabilitation Program for Enhancing Functional Outcomes and Participation in Children With Diplegic Cerebral Palsy: A Case Series

**DOI:** 10.7759/cureus.112057

**Published:** 2026-07-04

**Authors:** Sandeep Khanna, Ranganathan Arunmozhi

**Affiliations:** 1 School of Physiotherapy and Allied Health Sciences, Sardar Bhagwan Singh University, Balawala, Dehradun, IND

**Keywords:** case series, cerebral palsy, cp, function, parent-mediated home-based intervention, participation, pediatric physiotherapy, pediatric rehabilitation

## Abstract

Children with cerebral palsy (CP) often experience limitations in mobility, participation, and engagement in home, school, and play-related activities. Many children with CP are unable to access the required therapeutic services due to a variety of reasons, including financial constraints and caregiver availability. As CP is a lifelong condition, the required activities and exercises have to be repeated many times a day; a once-daily session with a therapist might be insufficient. Considering the comprehensive outcomes of therapy in lifelong conditions is significant to analyze the impact of an intervention program. Parent-mediated home-based rehabilitation programs may serve as a practical and possible approach to improve functional outcomes and participation of individuals with CP in daily life.

This prospective, non-consecutive case series aims to analyze the effect of a 10-week parent-mediated home-based rehabilitation program on participation, gross motor function, balance, functional independence, engagement, and functional outcomes in 10 children aged between 5 years and 12 years with diplegic CP. These children were classified at Gross Motor Function Classification System (GMFCS) levels II and III. The children presented with difficulties in independent mobility within their environment and reduced participation in day-to-day activities at home and in the community. The home intervention program was designed according to the needs and routines of each child. Parents were briefed at the onset of the program and provided hands-on training on how to conduct various tasks and exercises at home. Parents were supervised through two scheduled sessions during the intervention period of 10 weeks to ensure appropriate implementation of the program. To measure functional outcomes and participation, measures used included the Canadian Occupational Performance Measure (COPM), Functional Independence Scale for Children (WeeFIM), Goal Attainment Scale (GAS), Pediatric Balance Scale (PBS), Gross Motor Function Measure-66 (GMFM-66), and the Child Engagement in Daily Life Measure Version 2 (CEDL V2).

Mean scores demonstrated improvement in all outcome areas following the 10-week intervention. COPM performance improved from 3.1 to 4.2, while COPM satisfaction improved from 3 to 4.6. CEDL V2 frequency scores increased from 24.89 to 29.37, and CEDL V2 enjoyment scores increased from 23.3 to 25.8. WeeFIM scores improved from 72.5 to 78.5. The mean GMFM-66 scores improved from 63.12 to 64.84. GAS score improved from 40 to 44. Improvements were also seen in PBS scores, increasing from 33.3 to 35.3.

The findings suggest that the parent-mediated home-based rehabilitation may be an effective and practical intervention approach for enhancing functional outcomes and participation among children with diplegic CP wherever direct therapeutic services are not available. Further studies with larger sample sizes are recommended to establish long-term effectiveness and broader applicability.

## Introduction

Cerebral palsy (CP) is one of the most common childhood motor neurodevelopmental disorders, which affects movement, posture, and functional abilities [1]. Children with CP often experience impairments in maintaining balance, mobility, postural control, and participation in age-appropriate activities [2]. Oftentimes, parents may believe that therapy can only be effectively conducted by experts in a clinical setup or therapy room on a one-to-one basis, which may reduce the importance of home carryover, proper handling, mobility, and active engagement of the child at home [3]. Many children with CP are unable to access required therapeutic services due to financial constraints, limited availability of therapists, and caregiver difficulties in ensuring regular therapy visits. These limitations frequently affect the independence of individuals with CP within the home, school, and community, thereby reducing participation and engagement in meaningful daily activities [4,5].

Current rehabilitation approaches for children with CP increasingly emphasize interventions that are activity-based, participation-focused, and family-centered [6]. CP being a lifelong condition, parent-mediated home-based rehabilitation programs seem to be a practical strategy to promote continuity of therapy. This approach also aligns with the International Classification of Functioning, Disability and Health (ICF) model, thereby encouraging active caregiver involvement in the child’s rehabilitation process within the natural home environment and community [7]. This approach may improve accessibility to intervention, increase carryover at home by the caregiver, thereby providing more opportunities to repeat and practice tasks, hence targeting neuroplasticity to its maximum [8-10]. Therefore, the caregiver can learn proper handling and gain proficiency in integrating the therapeutic activities into the daily routines of the child, thereby improving motor planning.

Although this home-based parent-mediated approach is advocated by many health practitioners and interventionists, limited evidence is available on its effect on functional outcomes, participation, and engagement among children with diplegic CP in real-life settings. This case series aimed to evaluate the outcomes of a 10-week individualized parent-mediated home-based rehabilitation program in children with diplegic CP.

## Materials and methods

This 10-week prospective, non-consecutive case series was conducted at the Latika Roy Memorial Foundation, Dehradun, India, with 10 children from the physiotherapy department who attended the monthly follow-up program between August 2025 and October 2025.

The study included children with diplegic CP, aged between 5 years and 12 years, at Gross Motor Function Classification System (GMFCS) levels II or III, who were able to understand spoken commands and had a caregiver willing to participate in the home-based rehabilitation program. Children having any other neurodevelopmental disorders such as Down syndrome, metabolic disorders, and genetic conditions; medical conditions or recurrent illness including seizures, respiratory disorders, or dysphagia; history of orthopedic or neurological surgical procedures within six months before enrollment in the study; or enrolled in any other study were excluded from the study. 

Children with CP often present with multifaceted deficits and impairments that affect not just motor function but also activities of daily living, participation, communication, and social engagement. Therefore, evaluating the effectiveness of any rehabilitation program may require the use of a variety of outcome measures that evaluate the changes from broader perspectives. To evaluate impact, outcome measures used in this case series included the Canadian Occupational Performance Measure (COPM) [11], Functional Independence Measure for Children (WeeFIM) [12], Goal Attainment Scale (GAS) [13], Pediatric Balance Scale (PBS) [14], Gross motor function measure-66 (GMFM-66) [15], and the Child Engagement in Daily Life Measure Wersion 2 (CEDL V2) [16]. The details of the outcome measure used are given in Table 1. 

**Table 1:** Outcome measures used in the study Score ranges: COPM, Canadian Occupational Performance Measure (performance: 0-10; satisfaction: 0-10); CEDL V2, Child Engagement in Daily Life Measure Version 2 (frequency scaled score: 0-100; enjoyment raw score: 11-55); GMFM-66, Gross Motor Function Measure-66 (0-100); WeeFIM, Functional Independence Measure for Children (18-126); GAS (T-score), Goal Attainment Scale (30-70); PBS, Pediatric Balance Scale (0-56).

Outcome Measure	Details
Canadian Occupational Performance Measure (COPM)	Assessed parent-perceived performance and satisfaction in functional activities.
Children's Functional Independence Measure (WeeFIM)	Evaluated functional independence in self-care, mobility, and cognition.
Child Engagement in Daily Life Measures Version 2 (CEDL V2)	Assessed participation and engagement in daily activities.
Gross Motor Function Measure-66 (GMFM-66)	Evaluated changes in gross motor function.
Pediatric Balance Scale (PBS)	Assessed functional balance abilities.
Goal Attainment Scale (GAS)	Measured achievement of individualized functional goals.

Following recruitment, baseline assessments were conducted. Analysis of the children's needs on each outcome measure was carried out, and goals were set along with the parents. The home intervention program was designed according to the needs and routine of each child. Parents were trained at the onset of the program and provided hands-on training on how to conduct various tasks and exercises at home. Parents were supervised through two scheduled sessions at the 4th and 8th weeks during the intervention period of 10 weeks to ensure appropriate implementation of the home-based program. Parents' compliance with the routine was self-motivated, and they were not burdened by fixed exercise sets and repetitions. The intention was to let parents be more responsive to their child's wishes and involve them during the activities that happen at home naturally during the course of the day in active-assisted ways. Post-intervention analysis on all outcome measures was carried out after 10 weeks of the program. Descriptive statistics were used to summarise outcomes. Pre and post-intervention scores were compared to evaluate changes following the intervention. 

Informed consent was obtained from caregivers and parents before the study started. The study followed the Declaration of Helsinki principles. Approval was received from the institutional review board of Sardar Bhagwan Singh University (IRB No. SBSU/PhD/2020/PT-IRB/1010 (RIC) dated 6/8/2025). 

Participants were provided with a home-based parent-mediated rehabilitation program under supervision, designed to improve gross motor function, balance, participation, and functional independence in children with cerebral palsy. The program was formulated by the authors on established principles of task-oriented training, family-centered rehabilitation, and motor learning approaches. This intervention was individually tailored based on the child's motor abilities, GMFCS level, caregiver concerns, and functional goals established during the initial assessment. The rationale for individualization was to enhance participation in meaningful daily activities, improve adherence to the program and facilitate integration of therapeutic activities in the child’s daily routine. The program was implemented over a period of 10 weeks. Parents/caregivers were trained in a basic range of motion exercises and techniques of performing active-assisted activities at home as part of their daily routine by physiotherapists during monthly follow-ups, and were advised to continue the prescribed activities at home.

The instructions and hands-on training to parents by trained physiotherapists were aimed at correct methods for lifting, holding, seating, and assisting the child with mobility throughout the day. A customized program based on the daily routine was planned along with the parents of each child, with common guidelines. The guidelines provided were:

(1) Perform passive exercises for the feet, knees, and legs exactly as instructed (20 repetitions for each joint, daily); (2) perform stretching exercises for the calf, the front muscles of the hip, and the back muscles of the thigh (15 repetitions for each muscle, daily); (3) practice slowly rotating the torso to the right and to the left (10 repetitions in each direction, daily); (4) practice moving from a seated to a standing position in active-assisted ways (sit-to-stand exercises) (10 repetitions, twice daily); (5) play ball catching and throwing games with child; (6) at night, use a gaiter and AFOs as a night splint (if prescribed); (7) assist the child to stand, cruise along the wall or furniture and move from one place through self-initiated movements or active-assisted walking methods (use proper footwear while weight bearing activities); (8) under supervision, encourage the child to play football, cricket, and other outdoor games with other children in active, assisted ways; (9) involve the child in all daily living activities and provide assistance wherever necessary, such as using the toilet, brushing teeth, washing hands, bathing, dressing, eating meals, performing minor household chores, etc.; (10) gradually enhance the child’s contribution to all activities of daily living.

**Figure 1:**
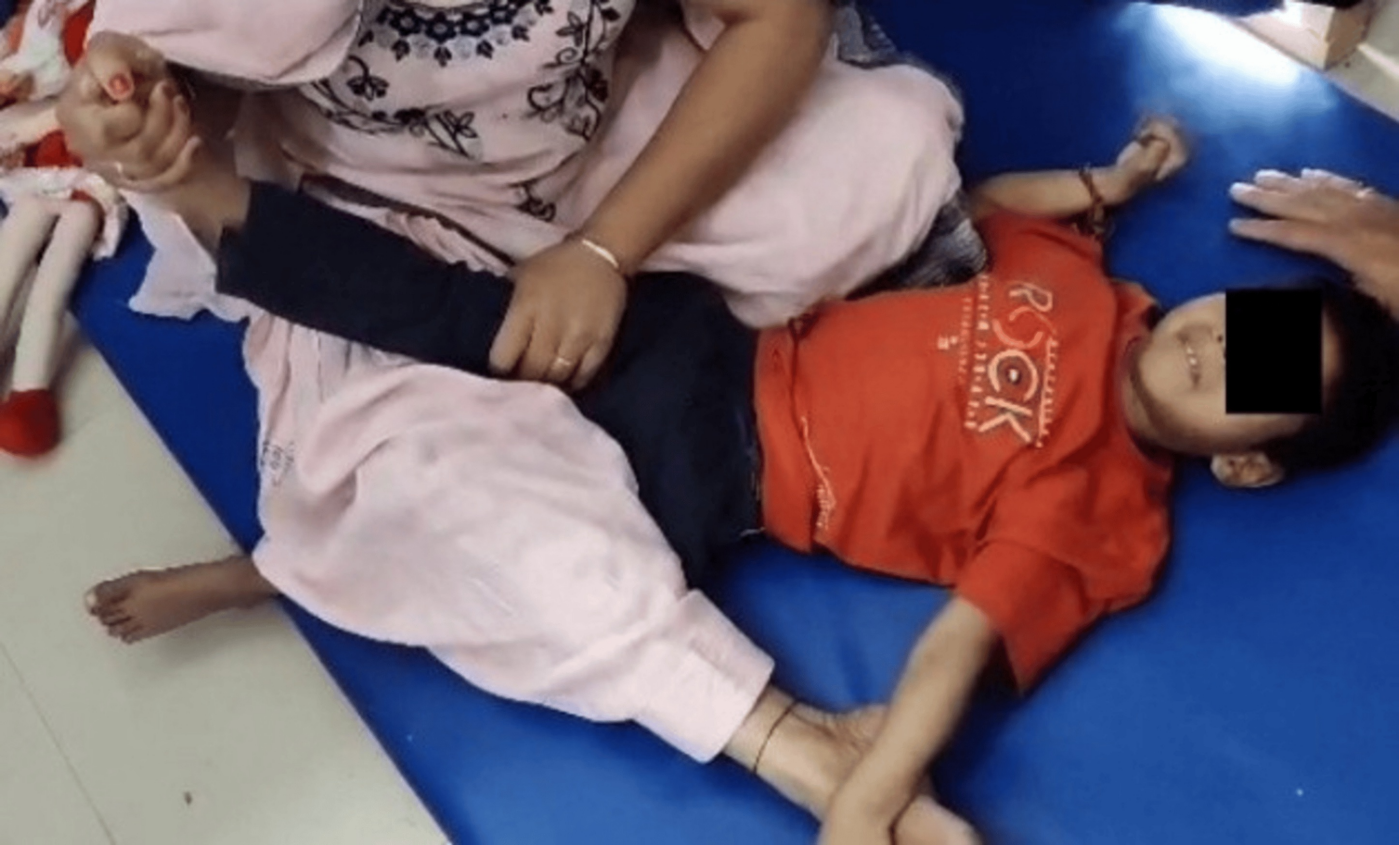
Stretching carried out by a caregiver

**Figure 2:**
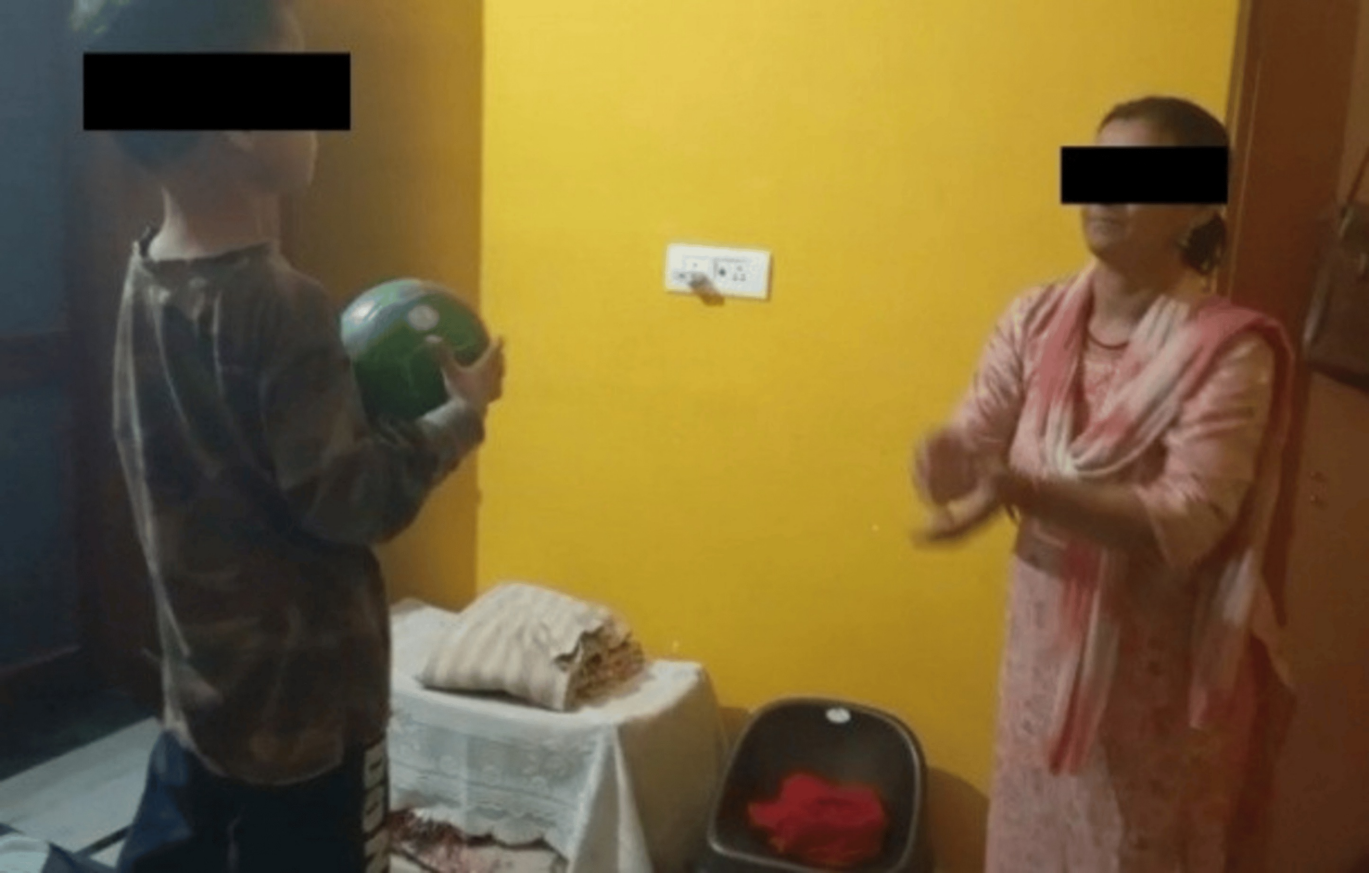
Caregiver playing catch and throw game with child

**Figure 3:**
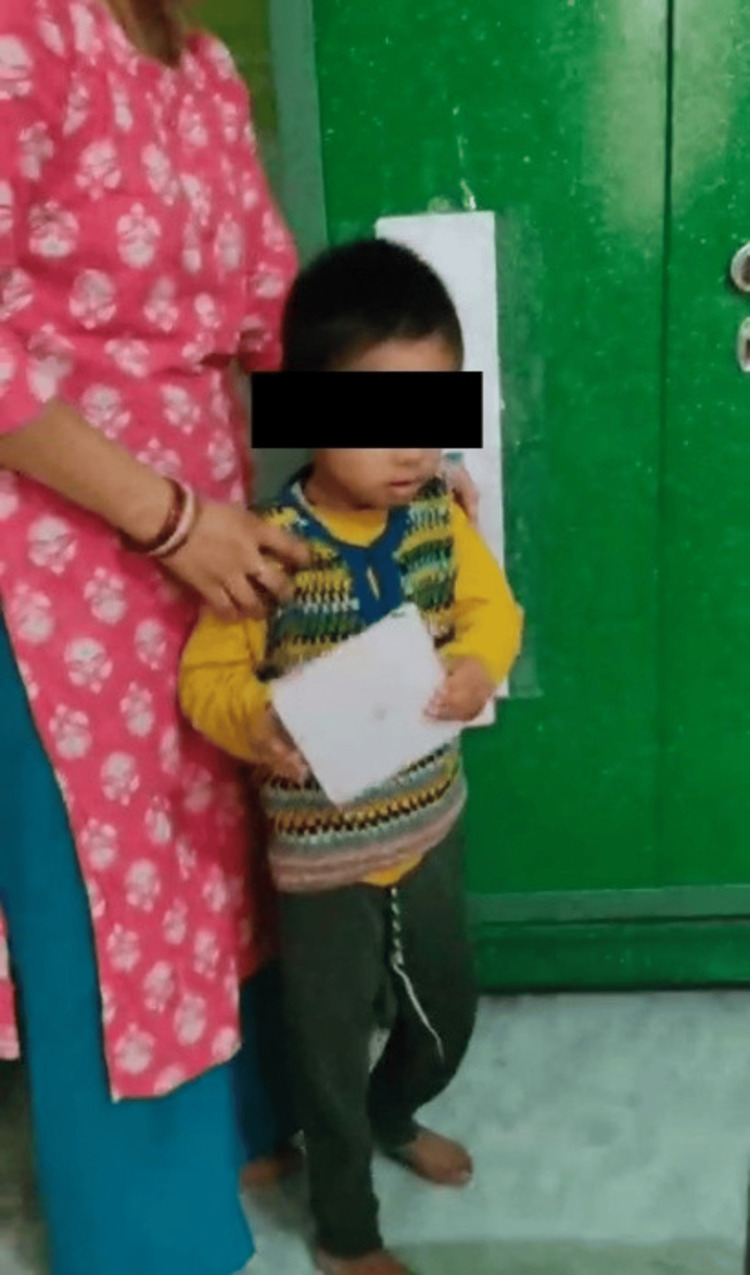
Caregiver assisting the child to walk at home

**Figure 4:**
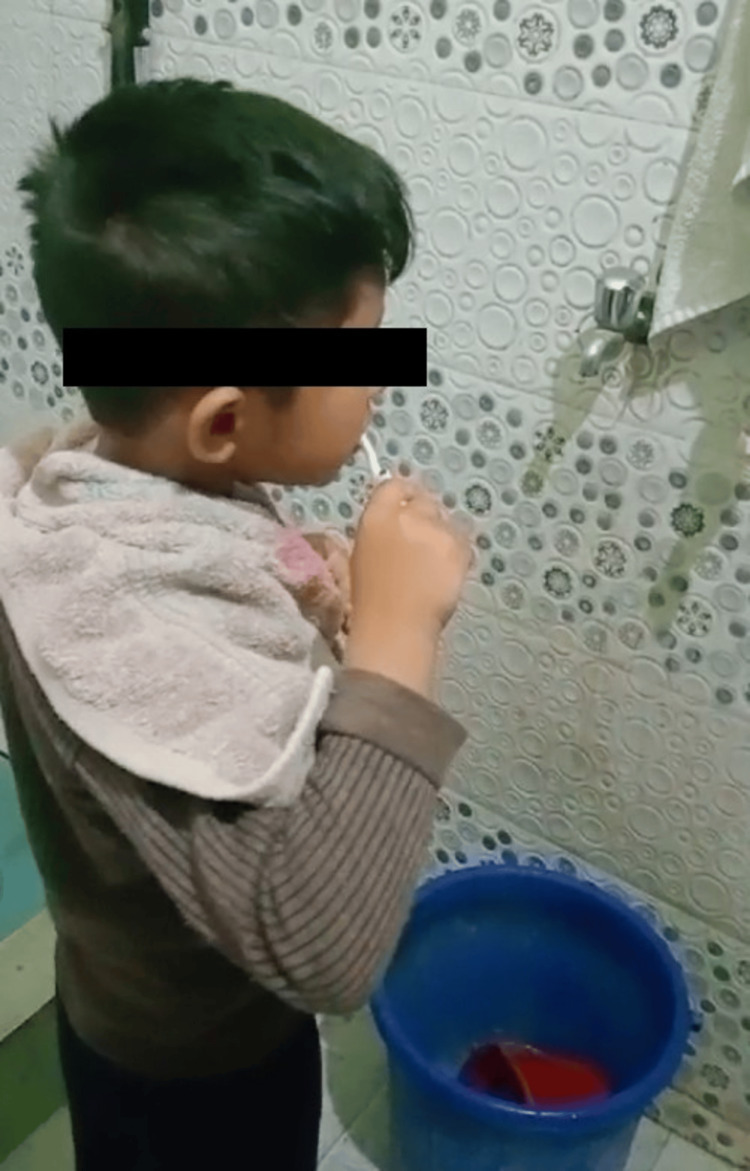
Child engaged in teeth-brushing activity at home

Figures 1-4 depict a few activities carried out by caregivers as per the guidelines.

## Results

Demographic details of the children included in the study are provided in Table 2, and Tables 3-4 present the pre- and post-intervention scores for all outcome measures. 

**Table 2:** Demographic details of the children

Patient	Age	Gross Motor Function Classification System (GMFCS) Level	Sex
Child 1	7 years 8 months	II	Female
Child 2	10 years 7 months	III	Female
Child 3	6 years 1 month	II	Female
Child 4	11 years 8 months	II	Male
Child 5	8 years 2 months	II	Male
Child 6	9 years 10 months	II	Male
Child 7	5 years	II	Male
Child 8	7 years 1 month	III	Male
Child 9	7 years 8 months	III	Male
Child 10	11 years 6 months	III	Female

**Table 3:** Pre-intervention findings on all outcome measures COPM: Canadian Occupational Performance Measure; CEDL V2: Child Engagement in Daily Life Measure Version 2; GMFM-66: Gross Motor Function Measure-66; WeeFIM: Functional Independence Scale for Children; GAS: Goal Attainment Scale; PBS: Pediatric Balance Scale

Patient	COPM		CEDL V2 (Part 1)		GMFM-66	WeeFIM	GAS	PBS
									Performance Score 1	Satisfaction Score 1	Frequency	Enjoyment
	Child 1	5	5	40.2					32	74.7	84	40	52
Child 2	3	2	25.7	26	54.1	65	40	8
Child 3	2	3	13.6	15	64.6	73	40	47
Child 4	3	2	25.7	24	72.2	67	40	51
Child 5	1	1	13.6	16	68.1	68	40	47
Child 6	6	5	43.5	35	65.6	71	40	43
Child 7	2	3	13.6	14	63	85	40	36
Child 8	1	2	21	20	53.6	67	40	11
Child 9	6	5	38.4	36	64	79	40	32
Child 10	2	2	13.6	15	51.3	66	40	6

**Table 4:** Post-intervention findings on all outcome measures COPM: Canadian Occupational Performance Measure; CEDL V2: Child Engagement in Daily Life Measure Version 2; GMFM-66: Gross Motor Function Measure-66; WeeFIM: Functional Independence Scale for Children; GAS: Goal Attainment Scale; PBS: Pediatric Balance Scale

Patient	COPM		CEDL V2 (Part 1)		GMFM-66	WeeFIM	GAS	PBS
									Performance Score 2	Satisfaction Score 2	Frequency	Enjoyment
	Child 1	7	8	46.5					34	76	88	40	54
Child 2	5	2	29.1	28	55.6	70	40	9
Child 3	2	4	13.6	17	65.3	78	40	47
Child 4	4	3	31.9	25	72.6	74	50	53
Child 5	3	2	21	19	68.5	75	50	49
Child 6	8	8	49.4	37	68.1	85	60	45
Child 7	2	4	13.6	16	65.3	91	40	39
Child 8	1	5	25.7	22	55.6	70	40	13
Child 9	8	7	41.9	40	67	83	40	35
Child 10	2	3	21	20	54.4	71	40	9

Following the parent-mediated home-based intervention program, an improvement was observed in the occupational performance and satisfaction scores reported by the caregivers. The mean COPM performance score increased from 3.1 ± 1.91 at baseline to 4.2 ± 2.65 post-intervention. Similarly, the mean COPM satisfaction score increased from 3.0 ± 1.49 to 4.6 ± 2.31 following the intervention. Many caregivers reported improvements in functional participation, mobility during daily routines, and engagement in home-based activities.

An improvement was observed in both the frequency of participation and the level of enjoyment during family and recreational activities. Following the intervention, the mean score for frequency of participation increased from 24.89 ± 11.97 to 29.37 ± 12.94. Similarly, the mean score for enjoyment increased from a baseline of 23.3 ± 8.62 to 25.8 ± 8.61 after the intervention. These findings suggest that, following the implementation of the home-based rehabilitation program, engagement and participation in daily living activities have increased.

The mean GMFM-66 score increased from 63.12 ± 7.90 prior to the intervention to 64.84 ± 7.40 following the intervention. The mean WeeFIM score increased from 72.5 ± 7.54 at baseline to 78.5 ± 7.76 after treatment. Caregivers reported improvements in activities related to self-care, mobility, and participation in daily tasks.

Goal attainment improved following the intervention. The baseline GAS score for all participants was 40. Following the intervention, the mean GAS score increased to 44 ± 6.99, indicating that children showed improvement in the individual functional goals established at baseline along with parents.

The mean balance score (PBS) increased from 33.3 ± 18.33 at baseline to 35.3 ± 18.18 post-intervention. Improvements were primarily observed in activities related to standing balance, transitional mobility, and dynamic balance.

Table 5 presents the mean ± standard deviation of the pre- and post-intervention outcome measures, along with the corresponding mean differences.

**Table 5:** Mean, standard deviation and mean difference of pre-intervention and post-intervention data COPM: Canadian Occupational Performance Measure; GMFM-66: Gross Motor Function Measure-66; WeeFIM: Functional Independence Scale for Children; GAS: Goal Attainment Scale; PBS: Pediatric Balance Scale

Outcome measure	Pre-intervention mean ± standard deviation	Post-intervention mean ± standard deviation	Mean difference
COPM-Performance	3.1 ± 1.91	4.2 ± 2.65	1.1
COPM-Satisfaction	3 ± 1.49	4.6 ± 2.31	1.6
Child Engagement-Frequency	24.89 ± 11.97	29.37 ± 12.94	4.48
Child Engagement-Enjoyment	23.3 ± 8.62	25.8 ± 8.61	2.5
GMFM-66	63.12 ± 7.90	64.84 ± 7.40	1.72
WeeFIM	72.50 ± 7.54	78.5 ± 7.76	6
GAS	40 ± 0	44 ± 6.99	4
PBS	33.3 ± 18.33	35.3 ± 18.18	2

## Discussion

This case series was aimed at assessing the effects of a parent-mediated home-based rehabilitation program on gross motor function, balance, participation, and functional independence in children with cerebral palsy. The mean difference between the scores of all outcome measures is indicated in Table 5. The results of the study revealed that following the intervention period, positive improvements were observed across outcome measures, including the COPM, CEDL V2, GMFM-66, WeeFIM, GAS, and PBS. The improvements observed in COPM scores indicate that caregivers perceived enhanced participation and satisfaction regarding the children's daily activities. Since the COPM is a participation-focused and individualized outcome measure, these results suggest that this intervention may have had a positive impact on meaningful functional goals, which were prioritized by the caregivers/ parents [17].

Improvements related to participation were reflected in the CEDL V2 scores. The increased frequency and enjoyment of participation suggest that by directly involving parents in the rehabilitation process, there is a chance of better integration of therapeutic activities into daily routines and family-based activities [18,19]. The improvement observed in GMFM-66 scores indicates positive changes in gross motor abilities. The improvements noted in tasks related to standing, transitional activities, and walking can be attributed to repeated task-specific practice within the home environment and increased opportunities for motor learning [20,21]. Functional independence also improved following the intervention, as evidenced by increased WeeFIM scores. Caregiver involvement may have fostered more opportunities for practice during activities of daily living, resulting in a better utilization of functional skills within regular household activities [22]. Although these findings in improvement have small quantitative values, they still indicate positive results. 

The observed increase in PBS score indicates an improvement in postural control and balance capabilities. Home-based practice comprising activities related to mobility, reaching, and balancing may have contributed to these improvements. Rehabilitation programs facilitated with parental involvement offer numerous benefits, such as increased therapy intensity, caregiver empowerment, greater contextual relevance of activities, and better integration of therapy into daily life [23]. Home-based rehabilitation can also help overcome barriers such as transportation issues, accessibility challenges, and limited time availability for clinic-based therapy. The findings of this study align with previous research emphasizing the importance of family-centered and participation-oriented rehabilitation approaches for children with cerebral palsy [24,25].

However, this study has certain limitations. The sample size in this study was small, and it did not include a control group. Furthermore, it did not involve a long-term follow-up. Therefore, these findings should be considered preliminary and interpreted with caution. To further investigate the effectiveness of home-based rehabilitation programs conducted with parental assistance in children with cerebral palsy, future studies featuring larger sample sizes, randomized controlled designs, and long-term follow-ups are recommended.

Clinical recommendations

As cerebral palsy is a multifaceted and lifelong condition, individualized parent-mediated home-based rehabilitation may be considered as an adjunct to other ways of offering therapy for children with diplegic cerebral palsy. Physiotherapists should look at a variety of outcome measures when working with children with cerebral palsy to work on various aspects of recovery. Therapists should provide structured caregiver training and offer timely supervision to facilitate integration of therapeutic activities into daily routines. A family-centered and participation-focused approach may enhance function, engagement, and participation of children with diplegic cerebral palsy in day-to-day activities, especially in settings where access to regular therapeutic services is limited. 

## Conclusions

This case series suggests that an individualized parent-mediated home-based rehabilitation program may improve participation, gross motor function, balance, goal attainment, and functional independence in children with cerebral palsy. The findings highlight the potential of incorporating rehabilitation activities into daily routines through active caregiver involvement.
